# Motor expertise modulates cortical activation during imagery of simple and complex actions

**DOI:** 10.1073/pnas.2515027122

**Published:** 2025-09-22

**Authors:** Jie Yin, Ke Yang, Chendi Zhou, Junhua Dang

**Affiliations:** ^a^School of Psychology, Beijing Sport University, Beijing 100084, China; ^b^Laboratory of Sports Stress and Adaptation of General Administration of Sport, Beijing Sport University, Beijing 100084, China; ^c^Institute of Social Psychology, School of Humanities and Social Sciences, Xi’an Jiaotong University, Xi’an 710049, China; ^d^Department of Surgical Sciences, Uppsala University, Uppsala 75105, Sweden

**Keywords:** motor imagery, neural efficiency, expertise

## Abstract

Motor imagery (MI) engages neural systems that overlap with actual movement and is widely used in sport and rehabilitation. Yet conflicting findings suggest that experts show either reduced (neural efficiency) or increased (vivid simulation) brain activation during MI. We hypothesized that task complexity moderates this effect. Using fNIRS, we compared experts and novices during MI of simple versus complex soccer actions. Experts showed lower activation for simple tasks but higher activation for complex ones, while novices showed the opposite pattern. These findings unify opposing accounts of expertise, revealing that expert brains adaptively scale simulation to task demands. This has practical implications for optimizing MI-based training across skill levels.

## Significance

Motor imagery (MI) is widely used in sports training and rehabilitation, yet how expertise and task complexity jointly influence its neural basis remains unclear. Using fNIRS, this study shows that expert soccer players display lower cortical activation than novices when imagining simple actions, consistent with neural efficiency, but greater activation for complex actions, reflecting enhanced simulation. These findings highlight a dynamic interplay between skill level and imagery demand in shaping motor system engagement. The results offer a neuroscience-informed framework for tailoring mental practice according to an individual’s expertise and the cognitive load of the task.

Motor imagery (MI)—the mental rehearsal or simulation of an action without overt movement—engages neural mechanisms overlapping with actual movement execution (1–3) and is extensively used to improve performance in sports and motor rehabilitation (4, 5). Although MI continues to gain prominence in both research and applied settings, a central challenge remains: How should training protocols be tailored across different levels of motor expertise? Progress on this question has been hindered by the fact that existing evidence on how expertise modulates brain activation during MI is highly inconsistent.

On the one hand, several studies have reported that experts exhibit weaker or more spatially restricted brain activation than nonexperts when imagining skills in their domain—a phenomenon known as neural efficiency (6–9). For example, elite archers mentally rehearsing an archery shot showed focused activation primarily in the supplementary motor area (SMA), whereas novice archers recruited a broader network including the premotor cortex, inferior frontal gyrus, basal ganglia, and cerebellum (10). On the other hand, contrasting evidence indicates that in certain contexts, experts demonstrate greater brain activation than novices during action simulation tasks (11–13). For instance, basketball players tasked with predicting the outcome of a free throw from a shooter’s movements showed higher activity in the inferior parietal lobule and inferior frontal gyrus compared to novices (11).

We propose that task complexity acts as a critical moderator that can reconcile these divergent findings. Specifically, we propose that experts will exhibit neural efficiency—i.e., reduced cortical activation—when imagining simple or well-practiced movements, but heightened activation for complex, skill-specific actions that fully engage their expert motor schemas. In contrast, novices are expected to show the opposite pattern: They may exert more neural effort for simple movements due to less automatized control, but exhibit reduced activation for complex, sport-specific actions—not because these tasks are easy for them, but because they lack the necessary mental representations to simulate them. In the absence of well-developed motor schemas, the relevant cortical circuits may remain underengaged, resulting in comparatively lower activation despite higher task demands.

To test these predictions, we conducted two MI experiments manipulating task complexity within the domain of soccer foot skills. Experiment 1 involved three simple actions: 1) kicking the ball with the inside of the right foot (a soccer-specific movement), 2) leg lifting (a lower-limb but nonsport-specific action), and 3) arm lifting (a nonfoot movement). Experiment 2 retained the kicking condition and added two complex, soccer-specific movement sequences that require coordinated foot control: 1) right-footed alternating dribbling and 2) figure-eight dribbling with the right foot.

**Fig. 1.**
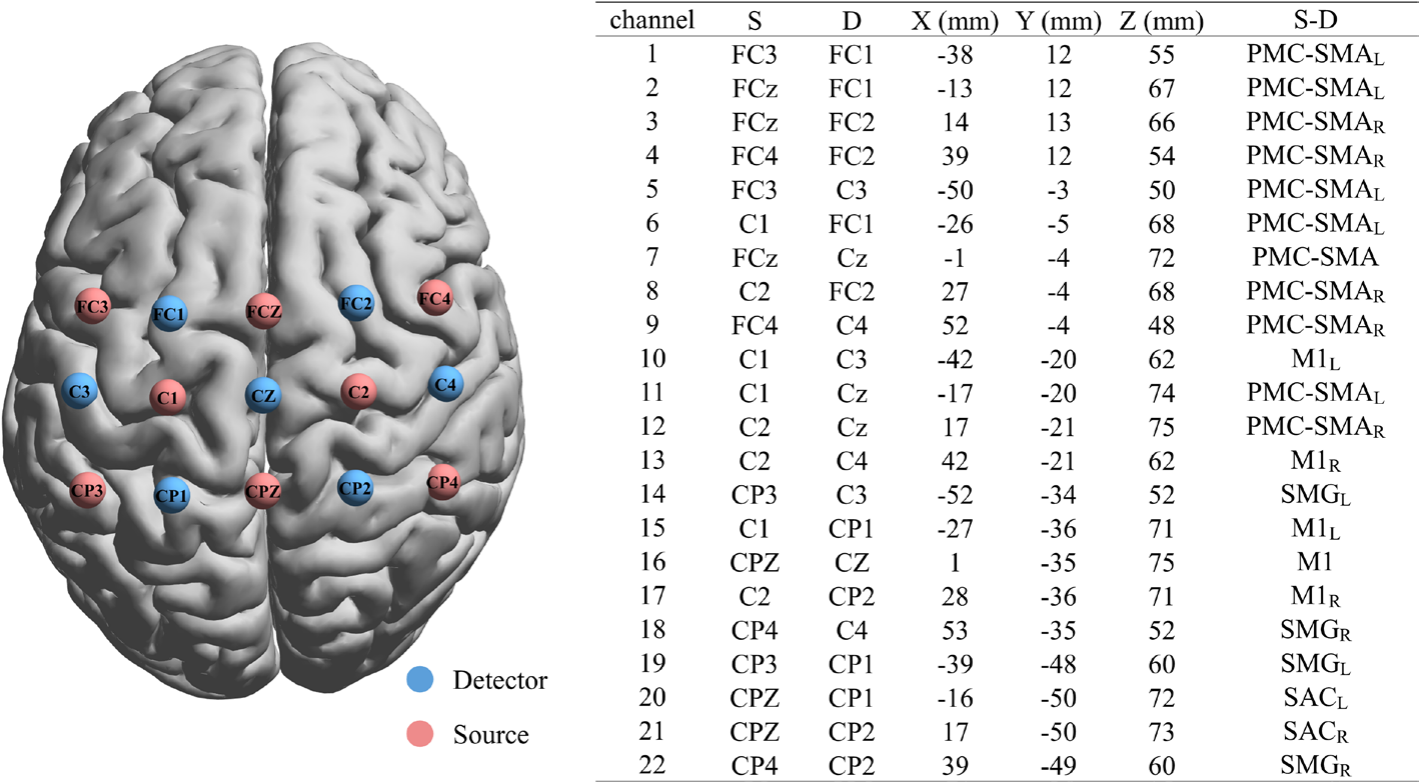
Brain map of the fNIRS probe set, including a table listing the specific brain regions covered according to the international 10 to 20 system. PMC-SMA: Premotor & Supplementary Motor Cortex; SAC: Somatosensory Association Cortex; M1: Primary Motor Cortex; SMG: Gyrus Supramarginalis; L: Left Hemisphere; R: Right Hemisphere.

Participants included skilled soccer players (experts) and individuals with no formal soccer training (novices). All participants performed MI tasks while we measured cortical activation using functional near-infrared spectroscopy (fNIRS). fNIRS optodes were positioned over key cortical areas implicated in motor planning and imagery, including the SMA, lateral premotor cortex, and inferior parietal lobule (supramarginal gyrus, SMG) bilaterally (Fig. 1). This optical neuroimaging method allows for noninvasive recording of oxygenated hemoglobin (HbO) changes from cortical surface regions. To rule out the potential confounding effect of individual differences in imagery ability, participants first completed the Revised Movement Imagery Questionnaire (MIQ-R) (14) prior to the MI task. This questionnaire assessed their general MI ability. After the MI task, participants rated the vividness and attentional engagement of their imagery using a 7-point Likert scale.

## Results

Repeated-measures ANOVAs were conducted on the mean and peak values of HbO across all 22 fNIRS channels, with movement as a within-subject factor and group (experts vs. novices) as a between-subject factor. False Discovery Rate (FDR) correction was applied to control for multiple comparisons, with significance set at *P* < 0.05. Since no interaction effects for peak HbO values survived FDR correction, we focus here on the mean HbO results.

**Fig. 2.**
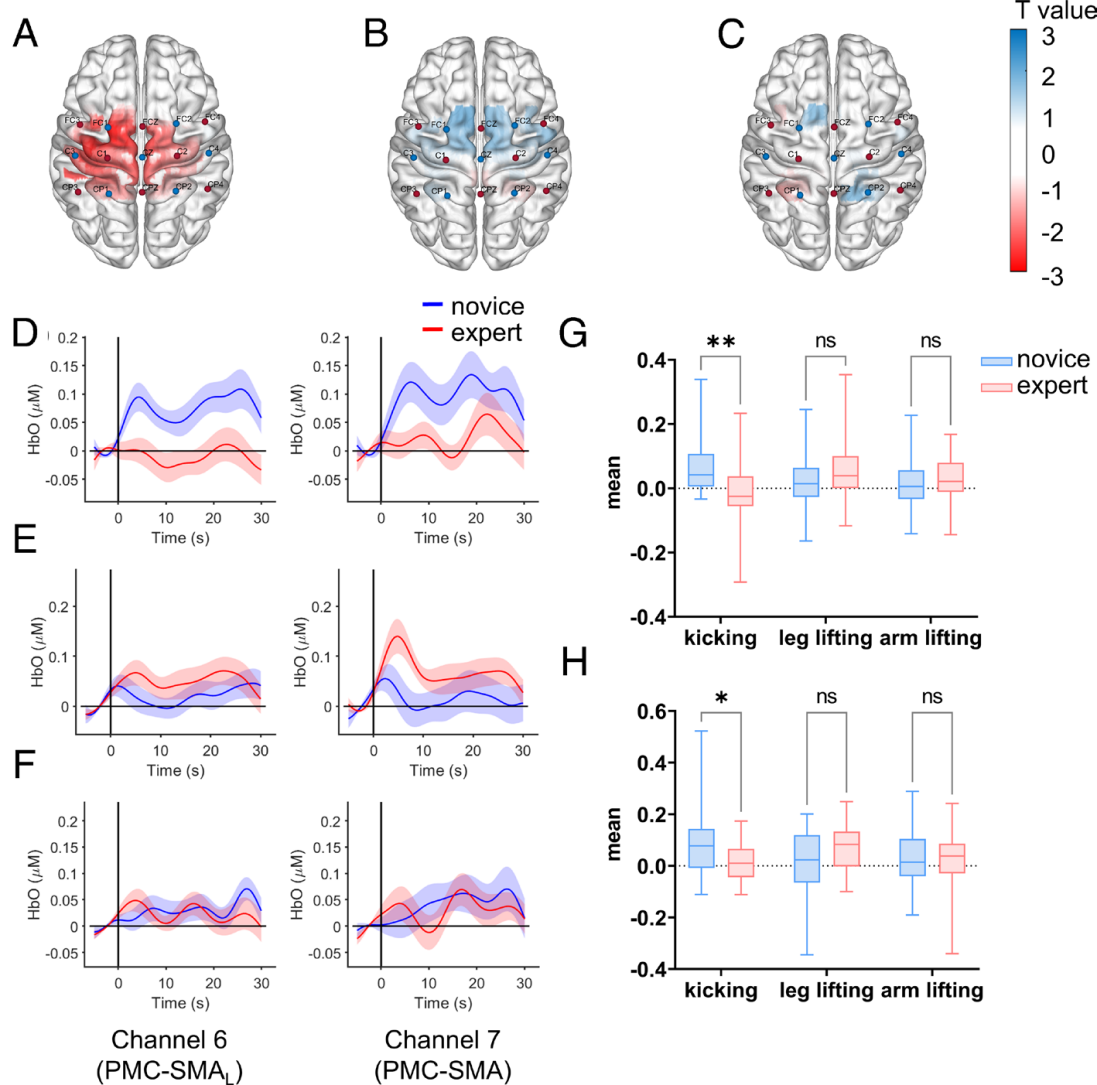
The *t*-statistic maps illustrate variations in HbO levels on the brain surface for kicking (*A*), leg lifting (*B*), and arm lifting (*C*). The functional graphs depict the temporal changes in HbO during three distinct movements: kicking (*D*), leg lifting (*E*), and arm lifting (*F*), recorded on channel 6 (*Left*) and 7 (*Right*). Shaded areas reflect the SEM. The mean HbO values obtained from channel 6 for the two groups during the imagery of the three movements are presented as (*G*), and from channel 7 as (*H*).

In Experiment 1, which examined imagery of simple movements (kicking, leg lifting, and arm lifting), significant movement × group interactions were observed on several channels, including channel 6 [*F* (2, 94) = 6.98, *P* = 0.001, η^2^_p_ = 0.129] and channel 7 [*F* (2, 94) = 7.33, *P* = 0.001, η^2^_p_ = 0.135], both of which remained significant after FDR correction (*p*s = 0.017). As shown in Fig. 2, simple effects analyses showed that, during imagery of the kicking action, experts exhibited significantly lower mean HbO than novices on channel 6 [*F* (1, 47) = 9.22, *P* = 0.004, η^2^_p_ = 0.164] and channel 7 [*F* (1, 47) = 6.00, *P* = 0.018, η^2^_p_ = 0.113]. No significant group differences were found for leg lifting or arm lifting on any channels.

Because channels 6 and 7 are spatially adjacent and both fall within the premotor–supplementary motor area (PMC–SMA), we conducted a region-level analysis by averaging their HbO values. The resulting composite ROI showed the same group × movement interaction pattern [*F* (2, 96) = 11.35, *P* < 0.001, η^2^_p_ = 0.191], with experts displaying significantly lower activation than novices during the kicking task [*F* (1, 48) = 11.64, *P* = 0.001, η^2^_p_ = 0.195]. No significant group differences were observed for leg lifting or arm lifting. These findings align with the channel-wise results and support the robustness of the observed effect.

In Experiment 2, which compared imagery of simple versus complex soccer-specific actions (kicking, alternating dribbling, and figure-eight dribbling), significant movement × group interactions were observed on multiple channels. After FDR correction, three channels retained significance: channel 1 [*F* (2, 84) = 6.07, *P* = 0.003, *p*_(FDR)_ = 0.029, η^2^_p_ = 0.126], channel 7 [*F* (2, 84) = 8.80, *P* < 0.001, *p*_(FDR)_ = 0.010, η^2^_p_ = 0.173], and channel 19 [*F* (2, 84) = 6.18, *P* = 0.003, *p*_(FDR)_ = 0.029, η^2^_p_ = 0.128].

**Fig. 3.**
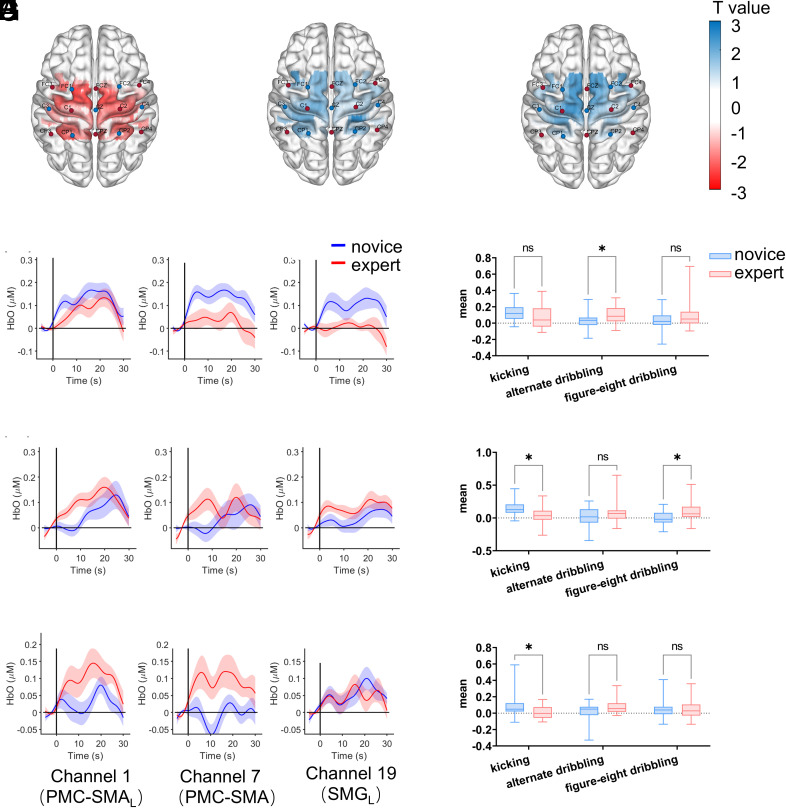
The *t*-statistic maps illustrate variations in oxygenated hemoglobin (HbO) levels on the brain surface for kicking (*A*), alternate dribbling (*B*), and figure-eight dribbling (*C*). The functional graphs depict the temporal changes in HbO during three distinct movements: kicking (*D*), alternate dribbling (*E*), and figure-eight dribbling (*F*), recorded on channel 1 (*Left*), channel 7 (*Middle*), and channel 19 (*Right*). Shaded areas reflect the SEM. The mean HbO values obtained from channel 1 for the two groups during the imagery of the three movements are presented as (*G*), from channel 7 as (*H*), and from channel 19 as (*I*).

As shown in Fig. 3, simple effects analyses revealed a crossover pattern: for the simple kicking action, experts again showed lower HbO than novices [channel 7: *F* (1, 42) = 6.18, *P* = 0.017, η^2^_p_ = 0.128; channel 19: *F* (1, 42) = 5.60, *P* = 0.023, η^2^_p_ = 0.118]. In contrast, for complex movements, experts showed greater activation. For example, on channel 1, experts exhibited higher HbO than novices during alternating dribbling [*F* (1, 42) = 5.32, *P* = 0.026, η^2^_p_ = 0.112], and on channel 7 during figure-eight dribbling [*F* (1, 42) = 5.87, *P* = 0.020, η^2^_p_ = 0.123].

In both experiments, there were no significant differences in general MI ability, as measured by the MIQ-R, between expert and novice participants. Similarly, no significant movement × group interactions were observed for the vividness or attentional engagement ratings of the imagery task.

## Discussion

This study reconciles a long-standing contradiction in the MI literature—why experts sometimes show reduced brain activation (neural efficiency) and other times show greater activation (enhanced simulation). By systematically manipulating task complexity, we demonstrated a crossover pattern: Experts exhibited reduced cortical activation during simple MI but greater activation during complex, domain-specific MI, while novices showed the opposite.

These findings suggest that the relationship between expertise and neural recruitment is not linear, but depends critically on task demands and the availability of internal motor representations (15). For simple, overlearned actions, experts likely rely on streamlined neural pathways, recruiting only the essential nodes required for accurate imagery. This economy of activation reflects years of fine-tuning and is consistent with the neural efficiency hypothesis (7, 8). In contrast, when imagining complex, sport-specific sequences that engage the full breadth of their skillset, experts mobilize broader sensorimotor and spatial networks to generate vivid and detailed simulations. Prior research has shown that experts activate premotor, parietal, and somatosensory regions more strongly than novices when observing or predicting skilled actions (12, 13), consistent with the enhanced simulation account (16). Importantly, although subjective imagery ability can influence neural responses (17), we found no significant group differences in general imagery ability (MIQ scores) or in task-specific vividness and attentional engagement ratings. This suggests that the observed expertise-related neural differences are unlikely to be driven by differences in subjective engagement or baseline imagery capacity.

Beyond resolving a theoretical paradox, our findings offer practical implications for the design of MI interventions in both sport and rehabilitation contexts. Critically, they highlight that the effectiveness of MI is not solely determined by whether imagery is used, but by what is imagined and who is imagining it. In sports training, these results suggest that the complexity of imagined actions should be calibrated to the athlete’s current skill level. For novices, asking them to simulate complex or unfamiliar maneuvers may fail to sufficiently engage the motor system, as evidenced by their low cortical activation during complex MI tasks in our study. To foster engagement and build internal motor representations, MI practice for beginners should begin with simple, fundamental movements that can be vividly and concretely imagined (e.g., a basic kick or pass) and progressively increase in complexity as their physical and cognitive schemas develop. Importantly, this progression should be guided by measurable indicators rather than subjective estimation. Structured MI programs could incorporate portable neuroimaging tools such as fNIRS or EEG to verify that the intended motor areas are being effectively engaged during imagery.

For skilled or expert athletes, the practical message is equally clear: To generate a meaningful neural challenge, MI scripts should feature complex, high-fidelity simulations that go beyond routine skills. Our data suggest that vivid simulation—and thus cortical engagement—scales with task difficulty only when the relevant motor schemas already exist. Therefore, MI interventions for experts should avoid overly simple tasks that may no longer stimulate adaptive neural responses. Instead, they should include novel tactical sequences, combinations of skills, or difficult game scenarios (e.g., responding to an unpredictable opponent), thereby maintaining cognitive engagement and enhancing transfer to competition contexts. Coaches and sport psychologists could draw from these principles to structure periodized MI routines that evolve alongside physical skill development, ensuring the brain remains as challenged as the body.

Similar principles apply in motor rehabilitation. Patients recovering from injury or stroke can be conceptualized as “novices” relearning specific motor patterns. Early-stage imagery training should emphasize basic, goal-relevant movements (e.g., weight shifting, isolated joint motions) that patients can feasibly imagine, thereby avoiding frustration and maximizing motor-cortical engagement. As patients regain function, more complex MI tasks—such as navigating a room, climbing stairs, or performing instrumental activities of daily living—can be incorporated to promote broader neural recruitment and functional generalization. Clinicians can also use portable fNIRS systems, as demonstrated in this study, to monitor cortical responses and support patients in optimizing their imagery—for instance, by introducing kinesthetic cues or guided scripts when additional support is needed to elicit sensorimotor activation.

In sum, the relationship between imagery, neural activation, and expertise is not fixed but responsive to both *task demands* and *individual capacity*. By matching MI content to the user’s internal motor representations and monitoring cortical engagement where possible, practitioners in both athletic and clinical settings can enhance the impact of imagery-based interventions. Our study thus bridges the gap between cognitive neuroscience and applied practice, offering not only theoretical resolution but also a practical blueprint for skill acquisition and recovery.

Although fNIRS offers practical advantages for ecologically valid and movement-free MI paradigms, it has certain limitations in spatial resolution and depth sensitivity. Specifically, this technique cannot measure activity in subcortical structures or the cerebellum, both of which are implicated in motor control and simulation processes. As such, our findings reflect cortical surface activation patterns and may not capture the full extent of neural engagement during imagery. Future studies could combine fNIRS with high-resolution neuroimaging techniques such as fMRI to provide a more comprehensive understanding of both cortical and subcortical contributions to motor expertise.

In addition to spatial activation patterns, recent studies emphasize the temporal dynamics of MI as a critical dimension of expertise. For instance, Guillot et al. demonstrated that fast-paced imagery—where imagined movements are deliberately sped up—preferentially activates cortical sensorimotor circuits in experts, suggesting that simulation speed may index the functional efficiency and plasticity of internal motor representations (18). Although the current study adopted natural-speed imagery to reflect typical usage in sports and rehabilitation settings, future research could benefit from manipulating imagery speed or imposing explicit temporal constraints to further characterize how experts flexibly adapt simulation timing to task demands. Such approaches may yield novel insights into the temporal precision of expert motor cognition and its neurophysiological correlates. Future research might also consider applying cluster-based permutation techniques to event-related designs with greater temporal resolution, which could offer a more integrated view of the spatial and temporal dynamics underlying MI.

## Materials and Methods

### Participants.

Participants in both experiments were recruited from Beijing Sport University through posted advertisements and in-class announcements. Interested individuals completed a brief screening to determine eligibility. The full study protocol, including all recruitment and informed consent procedures, was reviewed and approved by the Ethics Committee of the School of Psychology at Beijing Sport University. All participants met the following inclusion criteria: They had no history of neurological or psychiatric disorders, no head or foot injuries within the past six months, and were right-foot dominant, as determined by self-report and behavioral confirmation. For participants in the expert group, additional criteria included: 1) at least three consecutive years of systematic soccer training; 2) prior participation in university-level or higher-level formal soccer competitions; and (3) regular practice at a frequency of at least twice per week. Participants in the novice group were required to have no prior experience in organized or systematic soccer training.

Sample size was determined through a priori power analysis using G*Power for a 2 (Group: Expert vs. Novice) × 3 (Task) mixed-design ANOVA, focusing on the interaction effect. To detect a medium effect size (*f* = 0.25) with 95% power and α = 0.05, a total of 44 participants were needed. In Experiment 1, 50 participants (26 experts including 14 males, mean age = 19.65 ± 1.32 y; 24 novices including 11 males, mean age = 20.38 ± 2.60 y) were recruited to account for potential data loss due to motion artifacts or signal quality issues; however, no participants were excluded from the final analysis. In Experiment 2, 44 participants (23 experts including 7 males, mean age = 19.70 ± 1.26 y; 21 novices including 9 males, mean age = 20.24 ± 2.43 y) were recruited according to the power analysis, with no exclusions. The two experiments involved independent samples, with no overlap between participants in Experiment 1 and Experiment 2.

### General Procedure.

Each experimental session followed a fixed sequence. First, participants completed the MIQ-R, which was used to assess their general MI ability (14). The MIQ consists of eight simple motor actions. For each action, participants were instructed to imagine performing it and then rate the ease or difficulty of generating both visual and kinesthetic imagery on a 7-point Likert scale (1 = very difficult to see/feel, 7 = very easy to see/feel).

Following the MIQ-R, participants proceeded to the main MI task, during which fNIRS data were collected (see next section for details).

After completing the MI task, participants filled out a short questionnaire assessing participants’ subjective experience of the imagery task by asking them to rate the vividness and attentional engagement of their imagery, using a 7-point Likert scale (1 = very inactive/very inattentive, 7 = very vivid/very concentrated).

### MI Task.

**Fig. 4.**
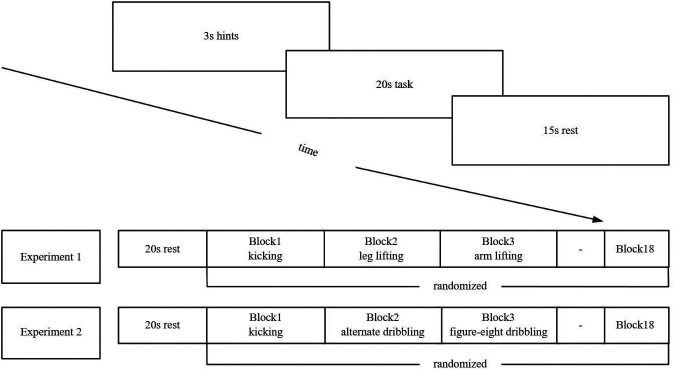
Schematic representation of the experimental procedure sequence in the two experiments.

Participants in each experiment completed three MI conditions using a block design implemented in MATLAB (2020b) with Psychtoolbox. Each block consisted of a 3-second cue phase (e.g., “kick”), a 20-s imagery period during which participants kept their eyes closed and engaged in kinesthetic imagery, and a 15-second rest phase with eyes open. Movement order was counterbalanced using a Latin square design to minimize order effects (Fig. 4). Before the task, all participants were instructed to use first-person kinesthetic imagery, imagining the movements as if they were physically performing them from their own perspective, focusing on the sensations associated with movement rather than visualizing themselves from an external viewpoint.

Participants performed three movement imagery tasks while standing: Experiment 1 included basic kicking, leg lifting, and arm lifting; Experiment 2 involved soccer-specific actions—kicking, alternating right-foot dribbling, and figure-eight dribbling. In Experiment 2, to ensure comprehension, participants were shown demonstration videos and completed nonball practice until they could mentally simulate each movement accurately. A brief practice session was included to standardize familiarity across participants.

### fNIRS Acquisition, Optode Configuration, and Preprocessing.

Cortical hemodynamic activity was recorded using a multichannel continuous-wave fNIRS system (N3001P Brainscan, Psyche-ark company, China) operating at three wavelengths (780, 808, and 850 nm) with a sampling rate of 20 Hz. Optodes were arranged according to the international 10 to 20 EEG system to target motor-related cortical regions: the SMA (centered around FCz/Cz), lateral premotor cortex (FC3/FC4), primary motor cortex (C3/C4), and supramarginal gyrus (CP3/CP4). Source–detector pairs formed 22 measurement channels with ~3 cm spacing.

Data were preprocessed using the Homer2 toolbox (v2.3) in MATLAB. Raw intensity signals were converted to optical density and bandpass-filtered (0.01 to 0.14 Hz) to remove physiological and instrumental noise. Motion artifacts were corrected using spline interpolation and wavelet-based algorithms. Hemodynamic concentration changes were calculated via the modified Beer–Lambert law with a differential pathlength factor (DPF) of 6.0. Block-averaged epochs were extracted from 5 s before to 30 s after imagery interval onset. Baseline correction used the –5 to 0 s preimagery interval. Mean and peak values of oxygenated hemoglobin (HbO) within the 0 to 30 s postcue window were computed for each trial and channel. HbO served as the primary activation index due to its greater signal-to-noise ratio.

## Data Availability

The data have been deposited in OSF (19).
